# Role of T cells in liver metastasis

**DOI:** 10.1038/s41419-024-06726-2

**Published:** 2024-05-16

**Authors:** Kejia Wu, Guozhu Zhang, Changbing Shen, Li Zhu, Chongyuan Yu, Kurt Sartorius, Wei Ding, Yong Jiang, Yunjie Lu

**Affiliations:** 1 Department of Hepatobiliary and Pancreatic Surgery, The First Affiliated Hospital of Soochow University, Suzhou, China; 2 Department of Hepatobiliary and Pancreatic Surgery, The Third Affiliated Hospital of Soochow University, Changzhou, China; 3 Department of Emergency Medicine, The Third Affiliated Hospital of Soochow University, Changzhou, China; 4 Department of Hepatobiliary and Pancreatic Surgery, Taizhou Second People’s Hospital Affiliated with Yangzhou University, Taizhou, China; 5 School of Laboratory Medicine and Molecular Sciences, University of Kwazulu-Natal, Durban, South Africa; 6 Africa Hepatopancreatobiliary Cancer Consortium, Mayo Clinic, Jacksonville, FL USA; 7 Department of General Surgery, Wujin Hospital Affiliated with Jiangsu University, Changzhou, China; 8 Department of General Surgery, The Wujin Clinical College of Xuzhou Medical University, Changzhou, China; 9 Changzhou Medical Center, Nanjing Medical University, Changzhou, China

**Keywords:** Metastasis, Liver cancer

## Abstract

The liver is a major metastatic site (organ) for gastrointestinal cancers (such as colorectal, gastric, and pancreatic cancers) as well as non-gastrointestinal cancers (such as lung, breast, and melanoma cancers). Due to the innate anatomical position of the liver, the apoptosis of T cells in the liver, the unique metabolic regulation of hepatocytes and other potential mechanisms, the liver tends to form an immunosuppressive microenvironment and subsequently form a pre-metastatic niche (PMN), which can promote metastasis and colonization by various tumor cells(TCs). As a result, the critical role of immunoresponse in liver based metastasis has become increasingly appreciated. T cells, a centrally important member of adaptive immune response, play a significant role in liver based metastases and clarifying the different roles of the various T cells subsets is important to guide future clinical treatment. In this review, we first introduce the predisposing factors and related mechanisms of liver metastasis (LM) before introducing the PMN and its transition to LM. Finally, we detail the role of different subsets of T cells in LM and advances in the management of LM in order to identify potential therapeutic targets for patients with LM.

## Facts

- Multiple factors lead to the formation of an immune-tolerant microenvironment in the liver, which is susceptible to tumor metastasis.
- LM is a complex pathological process. After entering the liver, tumor cells will first encounter a barrier composed of liver-resident immune cells which include T cells as an important player.
- The surveillance mechanism of the immune system can prevent tumor growth and invasion and understanding the transition to the PMN is a key step in LM that is closely related to immune tolerance.
- Different subsets of T cells play different roles in the process of LM, and understanding the mechanism is helpful to guide clinical work.

## Open questions

- The mechanisms of T cells in LM has not yet been fully clarified and requires further research.
- How can LM be ameliorated by harnessing T cell’s anti-tumor capacity and inhibiting the activity of immunosuppressive T cells?
- How can the balance between different subsets of T cells in LM be maintained?
- This paper mainly focuses on the role of T cells in liver metastasis and acknowledges the limitation that it does not consider other factors that may affect liver metastasis. These factors, for instance, include tumor type, stage, gene mutation, microorganism and metabolism.

## Introduction

Hepatocellular carcinoma (HCC) is the fifth most common malignant tumor in the world, with an increasing incidence and high mortality rate. Most HCC patients have progressed to advanced stage when diagnosed, and the prognosis is very poor, which seriously affects the quality of life of patients worldwide [1]. It is estimated that by 2040, more than 2 million people worldwide will develop liver cancer each year [2]. Strikingly, metastatic liver cancer is 18–40 times more common than HCC [3]. In addition to lymph nodes, the liver emerges as one of the organs most vulnerable to metastasis, particularly in cases of colorectal cancer where it is the primary tumor most likely to develop liver metastasis (LM). And LM stands as a significant contributor to mortality in gastrointestinal malignancies, melanoma, and breast cancer alike [4, 5]. According to statistics, if metastatic liver cancer is not well treated, the median survival of patients is typically only 6 to 12 months. The occurrence of LM is significantly associated with the decline of 5-year survival rate and quality of life of cancer patients. At present, surgical treatment is the most effective method [6, 7].

The immune system is an important weapon to defend against pathogens and harmful components, but abnormal immune responses can sometimes cause severe damage to the host [8]. In recent years, with the deepening of the biological research on liver cancer, there has been increasing interest in the relationship between metastatic liver cancer and immune system. Due to its unique anatomical location in the human body, the liver is considered an organ of immune tolerance, and various immune cells exhibit specific behaviors in the liver compared to other organs [9]. T lymphocytes are widely acknowledged as the primary defenders against cancer. Nonetheless, numerous studies have revealed that T lymphocytes can either promote or inhibit tumor growth, proliferation, and metastasis when stimulated by different factors. Therefore, understanding the role of T lymphocytes in liver-based metastases is crucial for understanding immunotherapy biology [10].

In summary, this review will introduce the basis of LM from the aspects of liver structure, the resident cell population of liver, and the formation of PMN. At the same time, we focus on the different functions and potential molecular mechanisms of various T-cell subsets in the progression of LM.

## Why is the liver a common site for tumor metastasis?

### Unique structural factors of the liver

Regarding the propensity of extrinsic tumors to metastasize to the liver, as early as 1889, Stephen Paget proposed the “Seeds and Soil” hypothesis. This hypothesis suggests that the affinity of tumors for the microenvironment can determine the site of metastasis [11]. Cancer cells, much like seeds, have the ability to disseminate throughout the body, yet they can only proliferate when they encounter fertile ground. The liver serves as such fertile soil. Contemporary scholars posit that a substantial portion of liver tumor metastasis can be attributed to its functional and anatomical factors, including the fenestrated regulation of hepatic sinusoidal endothelial cells and the immune-tolerant microenvironment [12].

The liver in the human body is positioned between the gastrointestinal tract and the systemic circulation. The human liver receives a blood flow of approximately 1.5 liters per minute. On one hand, the liver obtains blood from the circulation through the hepatic artery, and on the other hand, it receives blood from the gastrointestinal tract through the portal vein. The portal vein collects blood from the intestines, which is rich in various substances including metabolites, nutrients, soluble antigens, toxins, including endotoxins (such as LPS), among others. Consequently, the liver’s immune system must possess the capability of immune tolerance to cope with such complex external stimuli. Simultaneously, the liver’s immune system also needs to respond to various viruses, bacteria, parasites transmitted through the bloodstream, as well as secondary TCs that have migrated from other parts of the body. The presence of a multitude of cytokines, immunosuppressive cells, and ligands within the liver contributes to the establishment of a strictly immune-tolerant environment within the liver [13–15]. The immune-tolerant microenvironment exhibits a dual nature. On the one hand, it serves to prevent abnormal immune attacks on the host organism. On the other hand, to a certain extent, it provides a sanctuary for TCs, shielding them from immune cell-mediated destruction.

According to previous studies, both human and animal organ transplantation models, the liver shows strong immune tolerance, and the postoperative acceptance rate of allogeneic liver transplantation is ideal. As seen earlier in the classic porcine transplant model, despite major histocompatibility complex (MHC) mismatch between donor and recipient, The liver is indeed more receptive to allografts than other organs, and it has been observed in some models that the liver can maintain a stable state of the graft without immune suppression by external factors [16, 17]. In addition, rejection arising from transplantation of other tissues from the same donor can be incidentally suppressed by liver allotransplantation [18].

The blood transport system within the liver also plays a significant role in its immune regulatory functions. Terminal portal blood vessels, serving as the primary blood suppliers, contain a substantial population of lymphocytes. These lymphocytes interact within the liver sinusoids with liver-resident immune cells, thereby facilitating immune regulation. Liver sinusoidal endothelial cells (LSECs), as unique constituents of liver cells, lack tight intercellular connections and basal membranes. The endothelial barrier formed by LSECs is highly permeable, facilitating the exchange of substances [19]. Under normal circumstances, fenestrated LSECs have the ability to inhibit the activation of hepatic stellate cells (HSCs), thereby maintaining the homeostasis of the liver environment. However, TCs tend to induce LSEC defenestration, which leads to the induction of liver fibrosis and an increase in TC adhesion capabilities [20]. In the space known as the Disse space, situated between LSECs and blood vessels, various components such as Kupffer cells (KCs), dendritic cells (DCs), HSCs, and others are present. Due to its specific structural characteristics, including low perfusion pressure and limited space, circulating cells can reside in this space for extended durations. The Disse space provides an excellent growth environment for TCs, as it contains nutrient-rich filtrate from the liver sinusoidal blood flow without the interference or competition from other cells. Consequently, the development of metastatic foci within the liver often occurs more rapidly than in other locations [21]. Additionally, T cells can also be recruited to this area through chemotactic factors [22].

Due to the characteristics of the portal vein, various antigens delivered to the liver through the portal vein tend to induce tolerance, both locally within the liver and systemically throughout the body. This phenomenon is referred to as portal vein tolerance, and its mechanisms may involve antigen-presenting cells (APCs) participating in clonal deletion and the activation of regulatory T cells (Tregs), thus mediating immune suppression [23]. Research has indicated that the TLR4 signaling pathway is involved in CD8^+^ T-cell clonal deletion [24]. In the case of CD4^+^ T-cell clonal deletion, it may be closely associated with IFN-γ [25]. Furthermore, once activated, Tregs can induce immune tolerance by producing a series of immunosuppressive cytokines such as IL-10 and TGF-β1 [26]. The microenvironment of immune tolerance will lead to the decline of the function, number and distribution of cytotoxic cells, and instead promote immunosuppressive cells such as Treg, which eventually promote the metastasis and occurrence of tumors, and tumors can form a bad positive feedback loop by secreting inhibitory cytokines and exosomes [27].

### Apoptosis of T cells in the liver

The abnormal demise of T cells is intricately linked to hepatic immune tolerance. During the clearance phase of peripheral immunity, the accumulation of apoptotic CD8^+^ cells is often observed in the liver. Some researchers propose that hepatic immune tolerance is not a result of passive inactivation of T cells but rather is a consequence of active apoptosis. The mechanisms involved may be closely associated with KCs. KCs may induce T-cell apoptosis either directly by secreting FasL and nitric oxide or indirectly by stimulating NKT cells, which subsequently impact T-cell survival [28].

Researchers have conducted experiments by infusing the liver with a diluted cell suspension containing activated CD8^+^ T cells and activated CD4^+^ T cells, and the results indicated that the liver selectively retains CD8^+^ T cells [29]. The liver’s localization and apoptotic effects on activated T cells primarily target CD8^+^ T cells, while the impact on CD4^+^ T cells remains unclear.

Previously, researchers have reported the detection of a B220-expressing T-cell population by optimizing the isolation protocol of intrahepatic lymphocytes [30]. MacDonald et al. suggested that the expression of B220 on T cells serves as a marker for impending apoptosis [31]. This particular cell population exhibits functional similarities to the aberrant T cells abundant in Faslpr mice, which are mice with defective Fas death receptor expression. Such a defect often leads to dysfunctional apoptosis of activated T cells and eventually results in widespread T-cell involvement and immune system disorders [32].

Experiments have shown that injecting large quantities of antigen peptide into TCR Transgenic Mice can lead to substantial T-cell loss in peripheral blood. Surprisingly, T cells tend to accumulate and undergo apoptosis in the liver [33].To explain the accumulation of T cells in the liver, two possible mechanisms have been proposed. Firstly, the liver may recognize and sequester T cells that have initiated apoptosis or died in circulation [28]. Secondly, researchers believe that the liver has the ability to capture and destroy activated T cells, subsequently inducing apoptosis in these activated T cells. This phenomenon was originally referred to as “Responder Trap“ [34, 35]. During immune responses, activated T cells are recognized and trapped in the liver due to adhesion molecules, remaining sequestered until they lose recognition molecules and express B220, at which point they undergo apoptosis [35]. As for why the liver can recognize and capture T cells, it is suggested that adhesion molecules such as intercellular adhesion molecule-1 (ICAM-1), VAP-1, and antigen-specific recognition may play significant roles in this process [28].

Furthermore, studies have shown that in LM models, FasL-expressing CD11b^+^F4/80^+^ macrophages in the liver have the ability to siphon CD8^+^ T cells from circulation and can induce apoptosis in corresponding T cells through the Fas-FasL pathway. This process can lead to the formation of a systemic immune desert. Targeted therapy to eliminate immunosuppressive macrophages in the liver can successfully reduce the siphonage of liver macrophages and increase T-cell survival, effectively inhibiting the occurrence of LM [4].

### Immunoregulation by liver cells

From the perspective of human growth and development, the liver and bone marrow share several similar functions, with the most prominent being hematopoiesis and immunoregulation [36]. Hence, another plausible explanation for hepatic immune tolerance lies in the liver’s unique regulatory mechanisms. In contrast to cells in other organs, many liver-resident cells (whether parenchymal or non-parenchymal) possess antigen-presenting capabilities. What makes them even more unique is their ability to confer regulatory capacity upon circulating cells. Newly educated cells, in turn, can activate others, thereby inducing systemic immune tolerance, forming a complex yet intricate immunoregulatory system [8]. Apart from providing antigens to lymphocytes, APCs can also recruit lymphocytes from the circulating blood by presenting antigens. Hence, some have metaphorically likened the liver to a school, with APCs serving as teachers within this educational analogy [37–39]. Despite the presence of numerous APCs in the liver and their ability to activate T-cell immunity, liver APCs generally tend to induce immune tolerance. They often drive T-cell expansion but do not support T-cell cytotoxicity [40]. LSECs not only present endogenous antigens to CD8^+^ T cells but can also present exogenous antigens in the context of both MHC-I and MHC-II. In this manner, LSECs can activate both CD4^+^ and CD8^+^ T cells and induce tolerance to antigens from both blood and intestinal sources [41]. After antigen presentation by APCs, T cells can act immediately rather than following the more traditional Treg response. In the presence of PD-L1, activated CD8^+^ T cells do not develop into cytotoxic cells but instead exhibit an anergic state [42]. Apart from LSECs, other cells in the liver can also function as APCs. While liver cells are primarily involved in protein synthesis, metabolism, and detoxification of toxins, hepatocytes can recognize and present antigens from pathogens to the adaptive immune system in specific contexts [40]. Additionally, as one of the unique macrophages in the liver, KCs have been found to potentially promote immune tolerance by acting as anergic APCs. They may inhibit T-cell activation induced by APCs through the secretion of prostaglandins, making KCs another major factor in hepatic immune tolerance [43]. In general, most liver-resident cells, including KCs, LSECs, hepatocytes, etc., maintain an immune-tolerant state. This state may be attributed to factors such as the absence of co-stimulatory molecules, expression of IL-10, low expression of MHC, and others [44, 45]. An immune-tolerant microenvironment significantly increases the incidence of tumor metastasis, making the liver a high-risk area for tumor metastasis.

## The process of LM and liver-resident cell population

TCs often undergo a series of intricate mechanisms to eventually successfully migrate to the liver. This process is primarily divided into the following stages, collectively referred to as the invasion-metastasis cascade [46]. Initially, TCs acquire the ability to evade the primary tumor site due to morphological changes, such as the classic epithelial-to-mesenchymal transition (EMT). Subsequently, these cells can invade neighboring tissues through the extracellular matrix, followed by intravasation and extravasation, migration through endothelial cells, and ultimately settle in the target organ. The fate of metastatic TCs can include death, dormancy, or the formation of micrometastases on the liver after successful migration [47, 48]. From the perspective of vascular formation, the process can be primarily divided into five stages, including: PMN formation, micrometastatic phase, pre-angiogenic phase, angiogenic phase, and tumor growth phase [48].

After metastatic cancer cells enter the liver, they encounter an immune microenvironment composed of a highly specialized resident cell population. This population includes KCs, LSECs, HSCs, DCs. [49]. Circulating and bone marrow-derived lymphocytes are also recruited to the liver to respond to invading TCs [50]. When Circulating TCs (CTC) extravasate from circulation into the liver, LSECs, NK cells, and KC cells serve as the initial barriers encountered by the TCs and play crucial roles in the process of LM (51). Cancer cells trapped in the liver sinusoids can undergo physical damage due to mechanical stress-related trauma, and various immune cells can also contribute to the destruction and elimination of TCs through phagocytosis and cytotoxic functions [51].On the other hand, these specific liver-resident cells contribute to immune tolerance within the liver by secreting anti-inflammatory cytokines IL-10 and TGF-β, while also expressing PD-L1, which hinders T-cell activation.

LSECs represent a highly specialized subset of endothelial cells, constituting approximately 15–20% of hepatic cellular content. Their distinctive fenestra structure confers upon them a pivotal role in immune processes [52]. Under physiological conditions, LSECs’ fenestra structure imparts upon them a high endocytic activity, thereby contributing to the maintenance of hepatic intra-environmental homeostasis. Notably, studies have revealed that the pronounced endocytic activity of LSECs is efficacious in the removal of circulating autotaxin, an enzyme implicated in tumor metastasis and angiogenesis [53]. However, in the event of liver injury, LSECs undergo corresponding morphological and functional transformations, with the most conspicuous alteration being the loss of fenestrae and the formation of a basement membrane [52]. In the process of tumor metastasis, the interaction between LSECs and CTCs can alter the phenotype of both cell types, thereby determining whether TCs are cleared or continue to extravasate. It also plays a crucial role in the local microenvironment’s ability to form a metastatic niche [54]. Whether LSECs ultimately exhibit anti-tumor effects can be directly influenced by interactions with invading TCs or indirectly through the cytokines produced by KCs [54]. The release of Reactive Oxygen Species (ROS), Nitric Oxide (NO), and toxic free radicals by LSECs can lead to the death of TCs [55, 56]. LSECs can also induce T-cell tolerance to antigens originating from the liver sinusoids, thereby promoting immune tolerance to tumor-related antigens in the liver and ultimately facilitating TC metastasis [57]. It’s intriguing that endotoxin produced by gut bacteria can suppress the activation of CD4+ T cells by downregulating the expression of MHC-II, CD86, and CD80 on LSECs. This observation provides further evidence of the connection between immune tolerance and the distinctive structure of the liver [44].

NK cells represent a subset of innate lymphocytes with anti-tumor functions, constituting approximately 40% of liver lymphocytes. They exert their immune functions through signaling pathways such as TRAIL, FasL, and NKG2D [58]. The STING signaling pathway, mediated by NLRP3, can promote the secretion of IL-1β and IL-18 by macrophages. This, in turn, enhances the expression of 4-1BB in NK cells and 4-1BBL in macrophages, ultimately augmenting the anti-tumor activity of NK cells. Deficiencies in STING can exacerbate LM in mouse models and compromise the anti-tumor capabilities of NK cells [59]. Depletion of NK cells, whether through exogenous means or by using gene-targeting techniques, results in a significant increase in LM. Interestingly, different subpopulations of NK cells at various stages play dominant roles in different metastatic organs. For instance, immature NK cells mediate cytotoxicity via perforin in the liver, while mature NK subsets are more effective at reducing tumor burden in the lungs [60]. Indeed, emerging evidence suggests that NK cells not only participate in immune defense but also contribute to immune tolerance. One such mechanism involves NK cells inducing apoptosis in TRAIL-R2 expressing T cells Mediated by caspase-8. This selective elimination of specific T-cell subsets, rather than promoting inflammation, may aid in establishing immune tolerance, preventing excessive immune responses, and maintaining tissue homeostasis [61].

KCs are vital resident macrophages in the liver, constituting 80–90% of the body’s macrophage population [62]. They possess the capability to rapidly recognize, capture, and clear harmful substances such as TCs [63]. Research has shown that KCs can directly take up and clear CTCs, thereby inhibiting tumor metastasis in the liver, through mediation by C-type lectins (Dectin-2) and binding to Fc receptors [64, 65]. It has been observed that pre-administration of IFN-γ can enhance the anti-metastatic capabilities of KCs and NK cells in the early stages of LM [66]. However, intriguingly, some researchers have found that KCs play a bimodal role in the process of colorectal cancer liver metastases (CRLM). They act as inhibitors in the early stages of metastasis but exhibit contrasting effects in the later stages, potentially influenced by changes in VEGF and iNOS expression as well as T-cell infiltration [67].

HSCs are located in the Disse space between LSECs and hepatocytes, serving as the primary source of extracellular matrix (ECM) [68]. TCs can activate HSCs to facilitate a crucial step in LM-colonization. Moreover, HSCs not only promote angiogenesis to nourish tumors but also impair T-cell function, induce T-cell apoptosis and Treg expansion, thereby fostering immune tolerance [69, 70].

In summary, during the process of metastasis, TCs engage in complex interactions with parenchymal cells, non-parenchymal cells, and recruited T cells and other immune cells within the liver. On one hand, these cells possess the remarkable capability to eradicate tumor cells. However, their significance lies not only in their anti-tumor activity but also in their ability to foster immune tolerance through interactions with T cells. The central focus of my review is to explore strategies aimed at inhibiting liver metastasis by directly targeting T cells or indirectly influencing T cells through the stimulation of other immune/non-immune cells. In my previous review, I provided a comprehensive summary of the role played by Treg cells in liver fibrosis [71]. Building upon that foundation, this review will explore another liver disease that shares a contextual connection with this regulatory mechanism, I believe that a deep understanding of these regulatory mechanisms may offer new insights into preventing tumor metastasis to the liver.

## The formation of PMN in liver and its relationship with tumor metastasis

The term “PMN” was first introduced in 2005 and is typically used to describe the microenvironment of secondary tumor organs. Primary tumors release various cytokines that make the liver microenvironment more susceptible to the migratory growth of TCs and easier for TC colonization [72, 73]. Three key factors contribute to the formation of the PMN: the local microenvironment of the metastasis site, tumor-derived components, and tumor-mobilized Bone Marrow-Derived Cells (BMDCs) [74].

The liver is the largest parenchymal organ in the human body. On one hand, its inherent metabolic functions and anatomical positioning determine an immune-tolerant microenvironment. Additionally, adaptive immunity plays a crucial role in recognizing self and eliminating non-self antigens. The liver is densely populated with T lymphocytes, hepatic parenchymal cells, and non-parenchymal cells, and interactions among these cells often induce adaptive immune resistance within the liver. This immune-tolerant environment can, in turn, disrupt the function of liver T cells through various mechanisms such as anergy, clonal deletion, senescence, exhaustion, and deviation, thereby maintaining a local immunosuppressive microenvironment [75].

A crucial step in tumor metastasis is when CTCs acquire disseminating capabilities, enter secondary or distant organ sites, and proceed with subsequent metastasis. An important determinant of whether TCs can successfully establish themselves in these sites is the influence of the local microenvironment where CTCs reside. Primary tumors can induce the formation of the PMN, thereby creating a pro-tumor microenvironment and promoting tumor metastasis. The liver, as a typical immune-tolerant organ, is particularly conducive to the formation of the PMN [74]. Lu Chen and colleagues discovered that secreted Glucose Regulatory Protein 78 (sGRP78) released by TCs can interact with macrophages and dendritic cells (DCs) in the liver. sGRP78 not only inhibits DC activation and promotes macrophage M2 polarization but also induces the production of TGF-β in the liver. In summary, sGRP78 promotes liver immune tolerance and shapes the tumor microenvironment (TME) to facilitate the establishment of secondary tumors [76].

Furthermore, although the specific mechanisms remain unclear, exosomes are considered another major factor in the formation of cancer-induced PMNs [77]. Exosomal miR-25-3p tends to transfer from colorectal cancer (CRC) cells to LSECs and regulates the expression of ZO-1, occludin, VEGFR2, and Claudin5 on LSECs through the transcription factors KLF2/4. This promotes vascular formation and permeability [78]. Vascular formation can facilitate the entry of TCs into the bloodstream while providing abundant nutrients and oxygen to the metastatic site. Metastatic tumors must develop their own blood supply to succeed in metastasis [79].

Primary TCs can modulate the tumor and its adjacent tissues, forming the TME. This environment not only promotes the growth of the primary tumor but also influences organs elsewhere, laying the groundwork for future tumor metastasis. The TME can induce the secretion of various cellular and molecular components through multiple pathways, such as soluble growth factors, chemokines, cytokines, extracellular vesicles, Treg cells, and BMDCs, creating an immune-suppressive environment that weakens the function of CD8^+^ T cells. This, in turn, promotes the growth, invasion of the primary tumor, and the establishment of the PMN [80]. The recruitment of BMDCs in the liver can alter the phenotype of LSECs, HSCs, and immune cells. A PMN dominated by activated HSCs, metastasis-associated macrophages (MAMs), cancer-associated fibroblasts (CAFs), and various pro-metastatic factors is an optimal site for TC metastasis [81]. Among them, myeloid-derived suppressor cells (MDSC) is one of the most influential cell populations in BMDC, and it is closely related to the metastatic progression. We will describe the interaction between MDSC and T cells during LM below.

## In the context of LM, T cells play a pivotal role

Four well-known T-cell subtypes include Cytotoxic T cells (CTLs), Helper T cells (THs), Regulatory T cells (Tregs), and Natural Killer T cells (NKT cells). These T-cell subtypes can be further classified based on surface markers into the CD4^+^ T-cell family and the CD8^+^ T-cell family. Activated CD4^+^ T cells can differentiate into various subsets, such as Th1, Th2, Th17, and Tregs, characterized by their secretion of distinct cytokines and immunomodulatory factors. These T-cell subsets play diverse roles in tumor immunity and various inflammatory diseases, including autoimmune disorders, asthma, and allergies [82]. CTLs are considered the primary effector cells responsible for direct TC killing and are a vital component of anti-tumor immunity [83]. The balance between CD4^+^ and CD8^+^ T lymphocytes is crucial for maintaining normal immune function. Disruption of the ratio between these two T-cell subsets can lead to the development of malignancies [84]. NKT cells, unlike conventional T cells, recognize lipids rather than peptides. NKT cell markers mainly include CD56 and CD16. Interestingly, the two subtypes of NKT cells, Type I and Type II, exert opposing roles. Type I NKT cells play a significant role in tumor immunosurveillance and anti-tumor immunity, whereas Type II NKT cells are associated with immunosuppression in the context of cancer [85–87].

### CTL

CTLs are capable of secreting cytotoxic molecules such as granular enzymes and perforin, as well as pro-inflammatory cytokines like IFN-γ, TNF-α, and IL-9. They also promote apoptosis of TCs by expressing death ligands, such as FasL. Consequently, CTLs are considered a crucial effector cell population directly responsible for killing TCs, making them a vital component of anti-tumor immunity [88].

MDSCs possess immunosuppressive characteristics and constitute a critical subset of immune cells within the TME. They interact reciprocally with CTLs, affect CTL activity through a variety of signaling pathways and play a significant role in immune responses. Furthermore, they are associated with the progression and metastasis of various cancer types [89, 90]. Tumors can promote the expansion of MDSCs by generating keratinocyte-derived chemokines(KDC), ultimately leading to increased tumor colonization. The mechanisms involved include reducing the cytotoxicity of CTLs against TCs, suppressing the proliferation and differentiation of T cells, and inducing the generation of Tregs [91]. MDSCs can inhibit the function of CTLs through pathways involving the production of NO and ROS. This disrupts T-cell receptors and enhances antigen-specific tolerance, thereby suppressing CTL function [92]. Additionally, MDSCs can reduce the infiltration of CTLs into tumors by nitrating CCL2 chemokines, thereby protecting TCs from clearance [93]. Moreover, MDSCs deplete L-arginine, which is necessary for T-cell proliferation, by secreting arginase I [94]. Lastly, in collaboration with other immunosuppressive factors such as TGF-β and IL-10, MDSCs can enhance their inhibitory effect on CTLs and promote the development of Tregs [95]. Another study has shown that primary CRC tumors can induce the accumulation of MDSCs in the liver through the S1PR1-STAT3-IL-6 signaling pathway, ultimately suppressing T-cell proliferation in the Peritoneal Mononuclear Cell population [96].Within the TME, other immune components such as CXCL10, CXCL9, and CCL5 can induce the infiltration of CD8^+^ cells into tumor tissues through binding to their respective receptors, leading to direct TC killing [97]. Additionally, cytokines produced by T cells, such as IFN-γ, can inhibit cancer cell metastasis [98].

Nielson et al. discovered that granulin secreted by MAMs can activate HSCs to differentiate into myofibroblasts that secrete periostin, thus maintaining an immunosuppressive microenvironment [99]. In the process of LM progression of PDAC, hepatic myofibroblasts (HMF) highly express PD-L1 and can effectively weaken the tumor-killing ability of CTLS. However, PD-L1/PD-1 axis is not the dominant mechanism leading to immunosuppression,Further investigations are warranted to fully elucidate the underlying mechanisms [100]. In addition, Valeria et al. found that granulin leads to decreased proliferation and functionality of CTL cells in liver metastases [101]. Elisa et al. also observed a similar phenomenon where macrophages can exclude CTLs from TCs and limit the effectiveness of anti-PD-1 therapy. The combination of PLX3397 (an inhibitor of colony-stimulating factor-1 receptor) with anti-PD-1 therapy demonstrated promising results in tumor suppression [102].

Tumor-derived exosomes have been recognized as initiators of immune escape in cancer. Recent research has found that exosomes also impact T-cell function. NKG2D, an activating cytotoxic receptor, plays a crucial role in cancer immunity, and its abnormal loss in cancer can lead to immune suppression. Exosomes originating from prostate tumors downregulate the expression of NKG2D on CTLs and NK cells, resulting in decreased cytotoxicity and inducing tumor escape, ultimately promoting tumor proliferation, invasion, and LM [103]. Vautrot et al. discovered that exosomes expressing the specific ligand PD-L1 can reduce CTL proliferation [104]. Chen et al. found a negative correlation between exoPD-L1 and T-cell activation and infiltration in LM, suggesting it as a prognostic marker [105]. Exosomes also influence the activation of CD4^+^ T cells, which will be discussed in the Th cells section.

From a biological perspective, the liver comes into contact with a variety of products and foreign microbial communities from the gut through the portal vein. Studies have shown that gut microbiota play significant roles in primary liver cancer and LM [106, 107]. F. nucleatum, a common member of the oral microbiome, has recently gained attention for its oncogenic properties [108]. Through the mediation of pro-inflammatory factors, it can weaken the cytotoxic anti-tumor functions of CTLs and NK cells, thereby promoting the metastasis and proliferation of CRC TCs [109, 110].

In a mouse HCC model, in vitro experiments have shown that epithelial cells and immune cells release IL-33, which can preferentially expand CD8^+^ T cells and induce the activation of CD4^+^ and CD8^+^ T cells in the spleen and liver, leading to enhanced CTL cytotoxicity and exerting anti-tumor immune effects [111]. However, the anti-tumor function of IL-33 in the context of LM remains to be studied. In an in vivo experiment in a mouse CRC LM model, researchers performed single-cell RNA sequencing on tumors and corresponding adjacent tissues. The results showed a significant increase in Matrix Gla Protein (MGP) in clusters of cancer cells in CRLM. MGP is a vitamin K-dependent protein initially reported as an inhibitor of ectopic calcification. The mechanism may involve MGP promoting the accumulation of intracellular free Ca^2+^ levels, promoting NF-κB phosphorylation, leading to upregulated PD-L1 expression, and ultimately causing CD8^+^ T-cell exhaustion in tumors, resulting in tumor growth and metastasis [112].

Interleukin-1α (IL-1α) is a constitutively expressed protein found in various cell types and belongs to the IL-1 family, sharing the same receptor, IL-1R1, with IL-1β [113]. Kazuhiko et al. discovered that overexpression of IL-1α can activate the proliferation of CTLs and enhance their cytotoxic activity. This not only inhibits the growth of lymphoma but also suppresses the occurrence of LM [114].

**Fig. 1:**
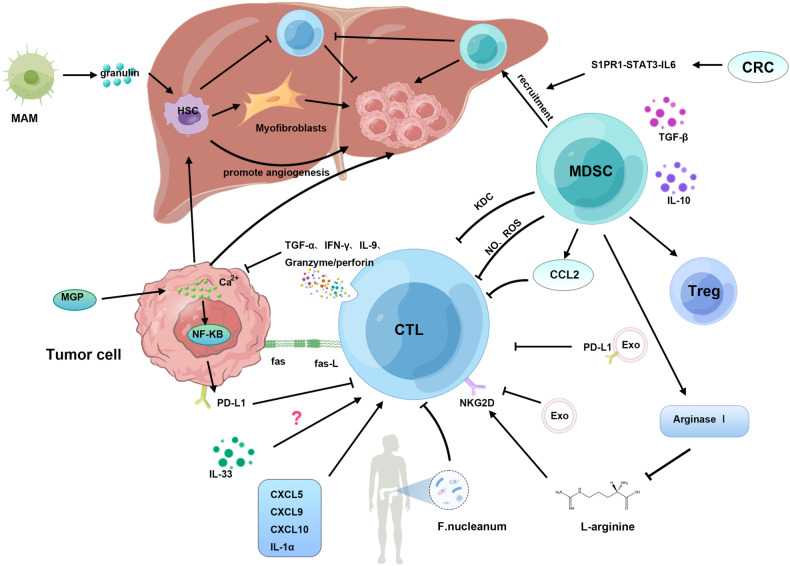
Regulatory mechanisms of CTLs. CTLs can secrete granular enzymes and perforin as well as a series of pro-inflammatory factors, and inhibit TCs by expressing death ligands. TC can generate KDC to expand MDSCs, thereby inhibiting CTL and promoting Treg. In addition, MDSCs can inhibit CTL through NO, ROS and CCL2 pathways. MDSCs secrete arginase I to reduce the requirement of L-arginine for T-cell expansion. The combination of TGF-β and IL-10 can enhance the cytotoxicity of CTL. Another study has shown that primary CRC tumors can induce MDSCs through the S1PR1-STAT3-IL-6 signaling pathway. Within the TME, CXCL10, CXCL9, and CCL5 induced CTL generation, and the overexpression of IL-1α also had a similar effect. Granulin secreted by MAMs can promote the differentiation of HSCs into myofibroblasts, thereby promoting immune tolerance in the tumor microenvironment. In addition, HSCs can also inhibit the generation of CTL. Prostate tumor-derived exosome down-regulates the expression of NKG2D on the surface of CTLs, resulting in a decrease in cytotoxicity. Exosome expressing PD-L1 can reduce the proliferation of CTLs. F.nucleanum could attenuate the cytotoxic anti-tumor function of CTLs. In HCC models, IL-33 potentiates CTL responses, but the role of IL-33 in LM remains unclear. MGP can promote the accumulation of intracellular free Ca2^+^ level and promote the phosphorylation of NF-κB, thereby activating and up-regulating the expression of PD-L1, leading to the exhaustion of CTL.

The entirety of the aforementioned signaling pathways is succinctly summarized in (Fig. 1).

### Treg

While T cells mostly play a role in tumor suppression, Tregs represent an immunosuppressive subset whose primary function is to hinder immune surveillance against cancer, prevent effective immune responses, and aid in tumor metastasis [115, 116]. TCs themselves can actively evade T-cell-mediated killing by producing and recruiting Tregs [117]. Tregs can inhibit the maturation of APCs, consume the immune factor IL-2 through the expression of the receptor CD25, and secrete suppressive cytokines through the expression of Granzyme and/or perforin, thereby clearing Effector T Cells and APCs [118]. Previous studies have demonstrated that Tregs can reduce the cytotoxic function of CTLs (116) and promote the colonization of metastatic tumors [119, 120].

Observations have shown that the infiltration of Tregs in advanced LM models is often associated with poor prognosis [121]. In the HBV-HCC continuum, HBV can induce the production of IL-8 through the MEK-ERK signaling pathway, and overexpression of IL-8 can significantly enhance intrahepatic metastasis by activating the IL-8-CXCR1-TGF-β signaling axis. Mechanistically, the selective induction of TGF-β by IL-8 leads to increased Treg polarization to suppress anti-tumor immunity [122]. James et al. discovered that LM can induce the generation of CTLA-4, PD-1, and ICOS+ Tregs. Tregs can modify tumor-specific MDSCs, causing them to migrate to distant tumors and inhibit the activation of tumor-specific CTLs through clonal anergy, thereby creating an immunosuppressive microenvironment (119). Molecular analysis indicates that in a liver tumor mouse model, genes encoding glucocorticoid-induced leucine zipper (GILZ) and CD83 are upregulated in Tregs, highlighting the importance of these encoded proteins in Treg immunosuppressive function [123]. The use of single CTLA-4 blockades or combination CTLA-4 and PD-L1 blockades can effectively reduce Treg cells within tumors [124].

CXCL16, one of the markers of cancer inflammation, plays a significant role in inducing the growth and metastasis of TCs, cellular communication networks within the TME, immune cell recruitment and differentiation, and angiogenesis. Research has found that CXCL16 is involved in the recruitment of Tregs and promotes their pro-tumor function. Interestingly, the concentration of secreted CXCL16 (sCXCL16) determines whether it promotes Treg growth, with sCXCL16 promotes Tregs at low concentrations and inhibits them at high concentrations [125]. Similarly, different doses of IL-2 can influence the generation of different T-cell types, with high doses of IL-2 stimulating and generating anti-tumor CTLs, while low doses of IL-2 mainly promote the development and growth of Tregs under resting conditions, ultimately contributing to the formation of immune tolerance [126, 127].

TNFR2 is highly expressed on the surface of certain TCs and immunosuppressive cells, particularly Tregs. It is a potential driver of immune evasion and tumor proliferation. Therefore, blocking TNFR2 represents an excellent strategy for cancer treatment [128]. Studies have found that the increase in TNFR2-expressing Tregs is associated with LM in lung cancer and colon cancer. Specific deletion of TNFR2 can significantly reduce the accumulation of Tregs and MDSCs, effectively reducing the extent of LM [119].

Tumor-Associated Macrophages (TAMs) have garnered attention due to their plasticity. Upstream cytokine signals determine whether TAMs differentiate into the tumor-suppressive M1 subtype or the immunosuppressive and tumor-promoting M2 subtype [129]. M1 macrophages can directly kill tumors by producing ROS and NO. On the other hand, M2 macrophages can induce the generation of Tregs and promote immune tolerance formation by secreting IL-10 and TGF-β. Additionally, M2 macrophages can promote tumor growth by inducing the production of polyamines and L-proline through fatty acid β-oxidation and the tricarboxylic acid cycle [130, 131]. KCs also exhibit similar functions by inducing Treg generation through IL-10 release and expressing PD-L1, thereby suppressing immune responses [132]. Shiri et al. revealed that Treg also act as the primary source of IL-10 secretion. Furthermore, IL-10 operates in an autocrine manner within Treg cells, boosting IL-10 secretion levels. This elevated IL-10 concentration exerts suppressive effects, resulting in reduced infiltration of CD8+ T cells and a weakened anti-tumor response in liver metastatic tissues. These effects are mediated by the up-regulation of PD-L1 expression in monocytes, thereby creating an immunosuppressive microenvironment that promotes tumor progression [133].

As mentioned earlier, MDSCs not only impact CTLs but also amplify Tregs. The expression of CD40 on the surface of MDSCs is not only significant for MDSC-mediated immunosuppression but is also necessary for the expansion of tumor-specific Tregs. Blocking the interaction between CD40 and CD40L between MDSCs and Tregs may provide a new direction for cancer immunotherapy [134]. HSCs can not only inhibit T-cell proliferation but also promote Treg expansion, possibly through the secretion of TGF-β. This creates an immunosuppressive liver microenvironment conducive to the metastasis of other tumors to the liver [70].

During local radiation therapy, a specific phenomenon known as the “abscopal effect” can occur. Local radiation therapy leads to the regression of distant non-irradiated metastatic lesions. Experimental evidence suggests a negative correlation between Tregs/effector T cells and the occurrence of the abscopal effect. Infiltration of PD-1+ T cells and Tregs may limit the abscopal effect of LM, affecting the effectiveness of treatment [115, 135].

**Fig. 2:**
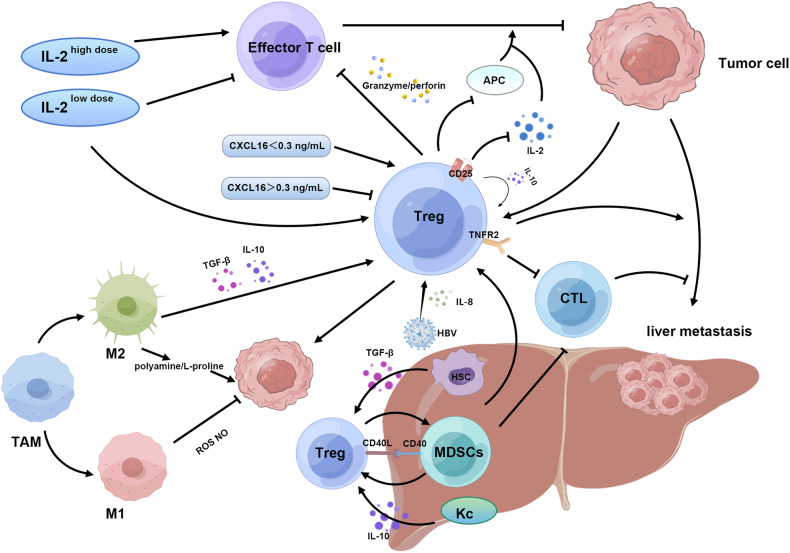
Regulatory mechanisms of Treg. TCs themselves can actively evade T-cell-mediated killing by generating and recruiting Tregs. Treg can inhibit the production of APC and immune factors, and reduce the cytotoxic function of CTLs. Different concentrations of sCXCL16 had different effects on Treg. Similarly, high doses of IL-2 promoted the generation of CTL, while low doses promoted the generation of Treg. The accumulation of Tregs and MDSC can be significantly reduced by specific deletion of TNFR2, which effectively reduces the extent of LM. The M2 subset of TAM can induce the generation of Treg cells and promote the formation of immune tolerance by secreting IL-10 and TGF-β. On the other hand, it can promote tumor growth through β-oxidation of fatty acids and tricarboxylic acid cycle. KCs can induce Treg generation by releasing IL-10 to promote immune tolerance. MDSCs and Tregs promote each other through CD40-CD40L. HSCs can amplify Treg cells by secreting TGF-β. Treg/ effector T cells were negatively correlated with the appearance of abscopal effect. Treg can secrete IL-10, which further promotes the release of IL-10 in an autocrine form and ultimately inhibits CTL. HBV can significantly enhance intrahepatic metastasis through the IL-8-CXCR1-TGF-β signal transduction axis.

The entirety of the aforementioned signaling pathways is succinctly summarized in (Fig. 2).

### Th cell

T helper (Th) cells play a crucial role in enhancing anti-tumor and anti-inflammatory immune responses by releasing various cytokines such as TNF-α, Granulocyte-Macrophage Colony-Stimulating Factor (GM-CSF), IL-2, IL-6, IL-10, IL-21, and others [82, 136].

Th cells can be categorized based on the different cytokines they secrete. Th1 cells primarily secrete cytokines like IFN-γ, TNF-α, IL-2, which promote the cytotoxic activity of macrophages and induce the proliferation and differentiation of NK cells and CTLs. Th2 cells secrete cytokines like IL-4, IL-5, and IL-13, which induce T-cell anergy and weaken T-cell cytotoxicity. However, Th2 cells enhance humoral immunity [137]. Therefore, some researchers consider Th2 cells as promoters of tumorigenesis and Th1 cells as suppressors of tumorigenesis [138]. LSECs in the liver can inhibit Th1 cells expressing IFN-γ and promote Th2 cells expressing IL-4, ultimately contributing to the formation of immune tolerance in the liver.

Pacheco et al. found that in liver cancer patients, there is a decrease in the number and function of Th1 cells, while the number and function of Th2 cells show an increasing trend. This imbalance in the ratio of Th1 to Th2 cells can lead to tumor metastasis [139]. Lucia et al. suggest that the ratio of anti-tumor Th1 cells to pro-tumor Th2 cells may have clinical significance in predicting cancer prognosis [140]. Kroemer et al. discovered that after surgery for CRLM, the expansion of CD4^+^ T cells is associated with poor prognosis [141]. This aligns with the findings of Katz et al., who observed a negative correlation between CD4^+^ T-cell content and post-CRLM surgery survival. The mechanism may be related to the polarization of CD4^+^ T cells in the liver toward Th2, resulting in the production of immunosuppressive factors [142]. In a similar lung metastasis model, Th2 cells have been shown to regulate macrophage phenotype and function to promote tumor metastasis [143].

TAMs, when differentiated into the anti-tumor M1 phenotype, can induce the generation of Th1 cells and enhance anti-tumor responses [131]. On the other hand, Th2 cells can promote the differentiation of macrophages into the M2 phenotype and suppress CTL proliferation by secreting cytokines like IL-13 and IL-5 [144]. M2 macrophages, in turn, release pro-angiogenic factors and growth factors (VEGF, b-FGF, IL-8), promoting a Th2-type immune response (140) and tumor metastasis [145, 146]. Philipp et al. found that increased activated Th cells tend to increase the cytotoxic activity of tumor-infiltrating T cells. This suggests that activated Th cells play a role in killing TCs by supporting the cytotoxic activity of T cells [147]. As mentioned earlier, exosomal miR-135a-5p, which can directly inhibit the activation of CD4^+^ T cells in a mouse CRC model, promoting immune tolerance and metastasis in the liver [148].

Another often overlooked aspect is the impact of surgical stress on the cytotoxic activity of liver mononuclear cells, ultimately leading to LM [149]. Therefore, regional anesthesia has been studied as a potential way to improve postoperative outcomes, and researchers have concluded that spinal block combined with sevoflurane general anesthesia, by maintaining the balance between Th1 and Th2 cells, enhances the suppressive function of liver mononuclear cells, thereby reducing the occurrence of LM [150].

Th17 cells, distinct from Th1 and Th2 cells, secrete cytokines such as IL-17, IL-21, IL-22, and IL-26 [151]. Th17 cells play a crucial role in autoimmune conditions and tumorigenesis, promoting inflammation in various pathological conditions. They can activate Th cells and DCs, induce and release multiple cytokines, maintain a chronic inflammatory state around the liver, create an inflammatory environment conducive to tumor growth, and ultimately form a microenvironment conducive to carcinogenesis [152]. Th17 cells have also been found to accumulate in various types of tumors, including lung cancer, CRC, melanoma, ovarian cancer, gastric cancer, and more [10].

The development and progression of cancer are closely related to inflammation. The term “double-edged sword” is often used to describe the dual role of immune inflammation reactions in the human body, where both appropriate and excessive inflammation can lead to drastically different outcomes [153]. When it applies to Th17, on the one hand, Th17 cells can secrete a large number of pro-inflammatory factors, make the liver in a long-term inflammatory state, and further promote tumor progression and metastasis. On the one hand, Th17 cells can ameliorate tumor metastasis by regulating immune cells and cytokines in the microenvironment of liver cancer [10]. Phosphatase and tensin homolog (PTEN) in Th17 cells can inhibit the IL-2 signal, downregulate the STAT5 and Treg signaling pathways, and upregulate a transcription factor, STAT3, which supports the Th17 pathway. Additionally, IL-23 further activates STAT3, RORα, and RORγt in Th17 cells, maintaining their chronic inflammatory environment, thereby promoting tumor formation [154].

Regarding tumor promotion, IL-17 secreted by Th17 cells can stimulate TCs through the IL-6/Stat3 signaling pathway, promoting their proliferation, migration, and invasion [155]. Furthermore, IL-17 can promote the function of MDSCs, inducing the formation of an immunosuppressive TME, and thus, promoting tumor development and metastasis [156, 157]. Li et al. found that IL-17A, a cytokine expressed by Th17, could promote tumor metastasis by activating the NF-κB signaling pathway to upregulate MMP2 and MMP9 expression [158]. Nicholas et al. found that IL-22, a cytokine expressed by Th17 cells, stimulates angiogenesis by activating ERK and Stat3 pathways and acts directly on endothelial cells to induce tumor angiogenesis, thereby promoting tumor metastasis [159]. In related studies, Zhang et al. discovered that Th22 cells also serve as a significant source of IL-22. Moreover, they found that Th22 cells play a pivotal role in promoting angiogenesis at the site of liver metastases through their mediation of IL-22 production [160].

**Fig. 3:**
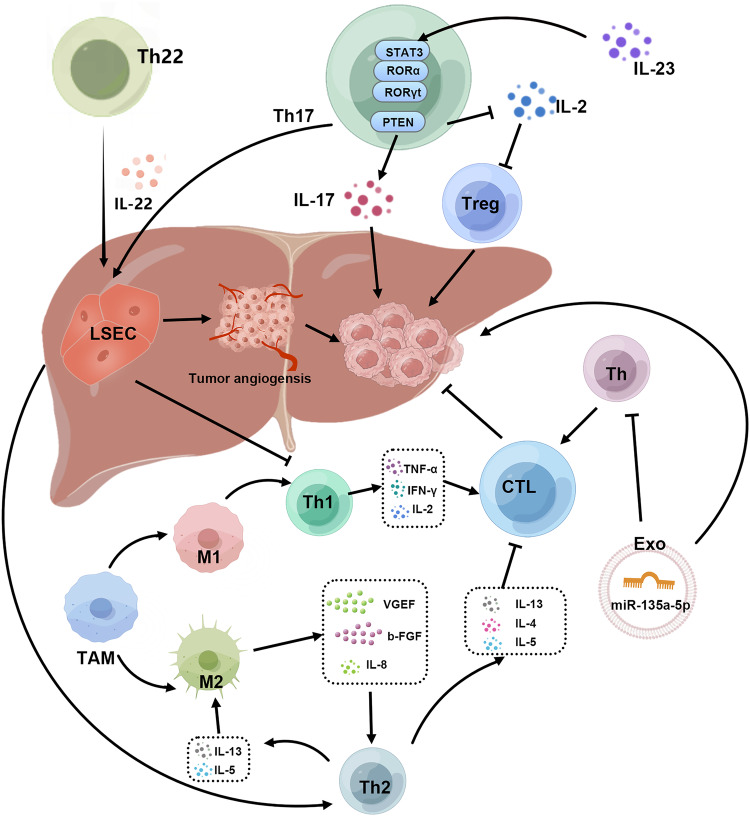
Regulatory mechanisms of Th cells. Th cells can be classified according to different secreted factors. Th1 cells mainly secrete IFN-γ, TNF-α, IL-2 and other cytokines, which play a role in inhibiting tumor. In contrast, Th2 cells secrete cytokines such as IL-4, IL-5, and IL-13 that induce T-cell anergic and attenuate T-cell cytotoxicity. LSECs in the liver were able to inhibit IFN-expressing Th1 cells and promote IL-4-expressing Th2 cells. The M1 subtype of TAMs can induce the generation of Th1 cells. In addition, Th2 cells can secrete cytokines such as IL-13 and IL-5 to promote the differentiation of macrophages into M2 type and inhibit the proliferation of CTL. Activated Th cells play a role in killing TCs by supporting the cytotoxic activity of CTLS. Exosomal miR-135a-5p could directly inhibit CD4^+^T-cell activation in a mouse CRC model by activating the LATS2-YAP-MMP7 signaling axis. On the one hand, Th17 cells can secrete a large number of pro-inflammatory factors, keep the liver in a long-term inflammatory state, and further promote tumor progression and metastasis. On the one hand, Th17 cells can affect tumor metastasis by regulating immune cells and cytokines in the microenvironment of liver cancer. Th17 can inhibit IL-2 production, and IL-23 further activates STAT3, RORα, and RORγt in Th17 cells to maintain their long-term inflammatory environment. IL-17 secreted by Th17 cells can stimulate the proliferation of TCs through the IL-6/Stat3 signaling pathway. Th17 cells stimulate angiogenesis by activating ERK and Stat3 pathways and act directly on LSEC to induce tumor angiogenesis, thereby promoting tumor metastasis. Th17 and Th22 can promote tumor angiogenesis by secreting IL-22.

The entirety of the aforementioned signaling pathways is succinctly summarized in (Fig. 3).

### NKT

NKT cells make up approximately 30% of lymphocytes in the liver [161]. They express αβ T-cell receptors and recognize lipid antigens presented by the MHC class I-like protein CD1d. Based on the expression of different T-cell receptors (TCRs), NKT cells can be divided into two subtypes, type I and type II. Type I NKT cells typically express a canonical semi-invariant T-cell receptor, while type II NKT cells have a diverse TCR repertoire [86].

Type I NKT cells play a role in anti-tumor immunity by producing Th1 cytokines such as IFN-γ and TNF, which recruit NK cells and CD8+ T cells, mediating anti-tumor functions. Additionally, type I NKT cells possess cytolytic activity and can directly lyse TCs expressing CD1d [162, 163]. α-Galactosylceramide (α-GalCer) is a ligand recognized by nearly all type I NKT cells, and it is also the agonist of type I NKT cells, through early or repeated administration of α-GalCer can effectively reduce the extent of LM in patients with CRC [86, 164]. Kobayashi explored the impact of IL-12 on primary and secondary tumor growth through a devised spontaneous liver metastasis model. The findings underscored that the anti-tumor efficacy of IL-12 primarily relied on conventional T cells, while its anti-metastatic potential was predominantly mediated by NKT cells [165]. Another related studies have revealed that IL-12 produced by DCs plays a crucial role in the activation of type I NKT cells by α-GalCer. The interaction between DCs and NKT cells may occur through the CD40/CD40L pathway. IL-12 promotes the secretion of IFN-γ by NKT cells, which, in turn, upregulates the expression of IL-12 receptors on NKT cells via autocrine signaling, ultimately enhancing the cytotoxicity of NKT cells [166]. Conversely, type II NKT cells have opposing effects compared to most type I NKT cells. They typically do not recognize α-GalCer and play a critical role in tumor immune suppression by promoting the generation and function of other immunosuppressive cells, such as regulatory Tregs and MDSCs [86]. NKT cells can produce IL-13, which has been identified as a tumor-promoting factor. IL-13 acts through the IL-4Rα–STAT6 signaling pathway to facilitate immune escape of TCs [167]. Further research has suggested that the mechanism underlying this effect may involve IL-13 inducing myeloid cells to produce TGF-β, which suppresses the immune function of CD8^+^ T cells [168]. Studies by Sadegh et al. have demonstrated that mice with NKT cell deficiencies exhibit significantly reduced melanoma LM compared to wild-type (WT) mice. Further investigation revealed that NKT cells, through MDSCs, produce IL-10 and induce increased expression of IL-10 receptors on NK cells, leading to decreased NK cell cytotoxicity and exacerbation of melanoma LM [85].

It has been observed that NKT cells express high levels of CXCR6 [161]. The gut microbiota has the ability to regulate bile acid metabolism and can increase IFN-γ production and promote the accumulation of NKT cells in the liver through the CXCL16-CXCR6 axis [169, 170]. Sodium butyrate (NaB), a major product of gut microbial fermentation, can effectively reduce LM in a CRC mouse model. Further analysis has revealed that NaB supplementation increases NKT cells and Th17 cells while reducing Tregs. This results in increased IL-17 production and decreased IL-10 secretion [171]. Moreover, blocking CXCL16 can induce LM from melanoma, but systemic activation of NKT cells can reduce the occurrence of metastasis [172].

Lipopolysaccharide (LPS), a common endotoxin and a component of the outer wall of Gram-negative bacteria, primarily consists of lipids and polysaccharides [173]. LPS preconditioning enhances the bactericidal activity of immune cells and reduces host inflammatory responses [174, 175]. Makoto et al. constructed an LPS preconditioning model by intraperitoneally injecting 5 μg/kg LPS daily for 26 days in mice. They found that LPS preconditioning effectively enhances the anti-tumor cytotoxicity of liver NK cells and NKT cells, reduces the expression of IFN-γ, and significantly decreases LM in a CRC model. Therefore, LPS preconditioning may become an important component of perioperative care for cancer patients [176].

**Fig. 4:**
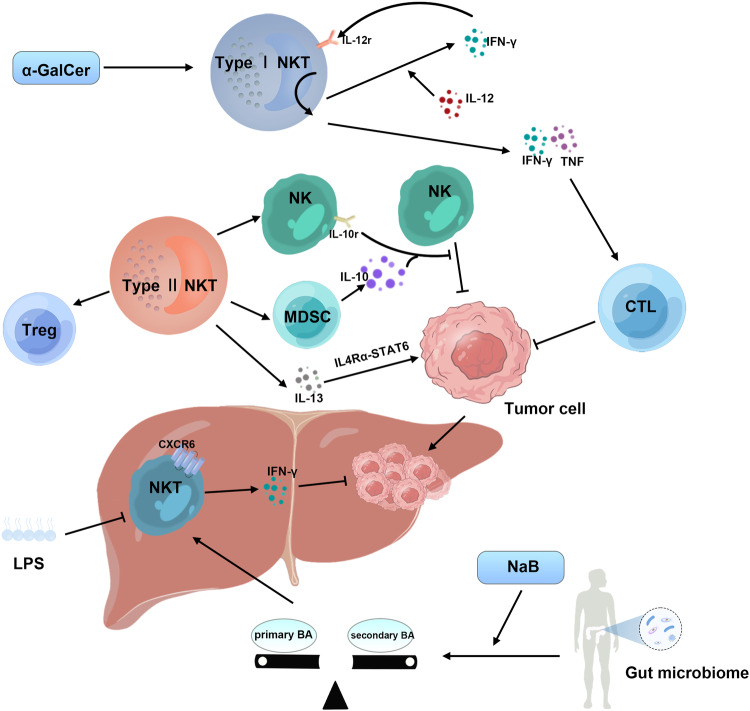
Regulatory mechanisms of NKT cells. NKT cells can be divided into two subtypes according to the different TCR expressed. Type I NKT cells secrete IFN-γ and TNF, recruit NK cells and CTL cells to mediate anti-tumor function. The use of α-GalCer can activate type I NKT cells to reduce the degree of LM in CRC patients. IL-12 can promote the secretion of IFN-γ by NKT, and IFN-γ can upregulate the IL-12 receptor of NKT in an autocrine manner, leading to the enhancement of the killing ability of NKT cells. Type II NKT does not normally recognize α-GalCer and plays a key role in tumor immune suppression. It can promote the generation of immunosuppressive cells such as Treg and MDSC. It can also secrete IL-13 and promote the immune escape of TCs through IL-4Rα-STAT6 signaling pathway. Increased production of IL-10 by NKT cells via MDSCs and induction of IL-10r expression by NK cells resulted in decreased NK cytotoxicity. Gut microbiota has the ability to regulate bile acid metabolism, which can increase IFN-γ production and promote the accumulation of liver NKT cells through the CXCL16-CXCR6 axis signaling pathway. NaB can increase NKT cells and Th17 cells and decrease Tregs, thereby increasing IL-17 and decreasing IL-10 secretion, thereby reducing the degree of LM. LPS preconditioning effectively enhanced the anti-tumor cytotoxicity of liver NK cells and NKT cells, reduced the expression of IFN-γ, and significantly reduced LM of colon cancer.

The entirety of the aforementioned signaling pathways is succinctly summarized in (Fig. 4).

## Advances in the treatment of liver metastases

For patients facing advanced metastasis, conventional surgical interventions seem carry significant risks. Currently, various non-surgical treatments for liver metastasis, such as ablation, chemotherapy, radiotherapy, and immunotherapy, exist; however, their efficacy is often constrained. Consequently, the pursuit of improved and safer treatment approaches has become increasingly imperative.

Radiofrequency Ablation (RFA) has emerged as a minimally invasive cancer treatment procedure that has seen rapid advancements in recent years. Through the introduction of radiofrequency electrodes, high-frequency electrical energy is directed to the target site, inducing thermal coagulation of tumor cells and triggering the formation of a reaction zone in the surrounding vascular tissue. In contrast to traditional surgical methods, RFA necessitates minimal tissue removal, offering enhanced safety and reduced incidence of complications. Consequently, RFA represents a viable option for patients ineligible for surgery or those experiencing postoperative recurrence [177, 178]. RFA not only achieves the physical inactivation of liver metastases but also generates a substantial amount of acute inflammatory signals in the form of necrotic cell debris. This process induces systemic immune responses, consequently mitigating the occurrence of immune evasion mechanisms. [179]. Furthermore, RFA has the capacity to induce the activation and maturation of DCs within the TME. This stimulation effectively promotes the formation of CD4+ and CD8+ T cells while augmenting the infiltration of effector T cells at the residual tumor site. However, tumor cells possess mechanisms to counteract the efficacy of effector T cells by up-regulating the expression of PD-1/PD-L1 and promoting the proliferation of Treg cells [180]. Hence, to address this challenge, researchers conducted experiments. In a mouse model of CRC, Shi et al. discovered that combining anti-PD-1 antibody immunotherapy with RFA effectively bolstered the T-cell immune response. This combination therapy not only substantially decreased cancer tissue volume but also inhibited distant tumor metastasis, thereby extending postoperative patient survival. [181]. Irreversible Electroporation (IRE) stands as a non-thermal ablation method employed in the treatment of locally advanced tumors. However, its efficacy as a standalone treatment is limited, often resulting in high rates of tumor recurrence. In response, Narayanan et al. investigated the therapeutic potential of combining IRE with a CD40 agonistic antibody (CD40Ab). Their findings revealed a significant reduction in the burden of liver metastases with the combined treatment. Besides, the infiltration of CD8+ cells, the activation and antigen recognition of DC, and the limitation of suppressive immune cells (Treg and MDSCs) were increased in liver metastases [182].

Low molecular weight heparin (LMWH) is commonly employed as an anticoagulant in clinical settings. However, it also possesses anti-tumor properties due to its ability to inhibit the formation of tumor neovascularization. This inhibition occurs through the targeting of vascular endothelial production factors and other pro-angiogenic factors, thereby impeding the formation and dissemination of tumors [183]. Quan et al. demonstrated that Low Molecular Weight Heparin (LMWH) has the capability to augment the infiltration of CD8+ T cells within liver metastases. Additionally, they observed that LMWH treatment normalized the vasculature within the TME. Notably, the combination of LMWH with immunotherapy exhibited enhanced anti-tumor activity compared to either treatment alone. This suggests that LMWH may potentiate the effectiveness of immunotherapy by promoting T-cell infiltration and modulating the tumor vasculature, thus providing a promising avenue for therapeutic intervention against metastatic tumors [184].

Appropriate chemotherapy holds the potential to eradicate primary lesions and liver metastases in CRC, thereby diminishing the extent of tumor spread. When combined with surgical intervention, this approach effectively enhances patient prognosis and survival outcomes [185]. However, Bosma et al. discovered that Oxaliplatin (OXA) treatment paradoxically facilitated tumor colonization and progression within the liver. Following OXA treatment, macrophages exhibited pronounced suppression of T-cell activation. Furthermore, there was a notable decrease in the proliferation, activation, and cytotoxicity of CD8+ T cells within the liver. Consequently, the utilization of OXA in the treatment of CRC patients warrants careful consideration and evaluation [186]. Combining chemotherapy with immunotherapy represents a burgeoning approach in cancer treatment. Due to the typically nonspecific symptoms associated with Pancreatic Ductular Adenocarcinoma (PDAC), liver metastasis is often present upon diagnosis, rendering chemotherapy alone less effective. Ho et al. conducted a study addressing this issue. Their findings revealed that concurrent administration of gemcitabine (GEM) and an anti-PD-1 antibody in a mouse liver metastasis model resulted in the expansion of T cells within metastatic tumor tissue. Furthermore, this treatment induced the polarization of macrophages into anti-tumor M1 cells. Additionally, there was a significant increase in CD8+ T cells within the TME, accompanied by enhanced intracellular expression of IFN-γ. Consequently, this regimen augments the capacity to eliminate tumor cells [187].

In the process of liver metastasis, CD11b+F4/80+ macrophages expressing FasL have been observed to siphon circulating CD8 + T cells and trigger their apoptosis via the Fas-FasL pathway, ultimately fostering an “immune desert” milieu. Targeting and eliminating these immunosuppressive macrophages in the liver presents a promising strategy. Building upon this premise, researchers have explored the potential of combining radiotherapy with immunotherapy to enhance treatment efficacy. Radiotherapy alone demonstrates the capability to diminish hepatic myeloid cell populations, reduce apoptosis of hepatic CD8+ T cells, and bolster the recruitment and survival of intrahepatic T cells, meanwhile, the ratio of CD11b+F4/80+ myeloid cells to CD8+ T cells is reduced. Furthermore, directional radiotherapy coupled with anti-PD-L1 therapy can stimulate CD8+ T cells to produce IFN-γ and granzyme B, thereby augmenting the liver immune response [4].

Ferroptosis, emerging as a research hotspot, represents a novel iron-dependent form of programmed cell death. Modulating the ferroptosis pathway, whether by activation or inhibition, has shown promise in mitigating disease progression, this offers a highly prospective strategy for treatment of a wide array of diseases [188]. Conche et al. have revealed that the GPX4-regulated ferroptosis of hepatocytes can trigger tumor-suppressive immune responses. Additionally, ferroptotic hepatocytes may release CXCL10 via the cGAS/STING pathway, thereby facilitating CD8+ T-cell infiltration into tumors. In a mouse model, administration of a triple combination consisting of a ferroptosis activator (Withaferin A), an immune checkpoint inhibitor (α-PD-1), and a myeloid-derived suppressor cell inhibitor (SB225002) yielded promising results. While this regimen exhibited limited efficacy against colorectal cancer itself, it notably diminished liver metastasis. This innovative therapy holds potential for combating liver metastasis across various cancer types. By employing this combination approach, the tumor is confronted on multiple fronts: firstly, T cells are stimulated to target cancer cells; subsequently, the inhibitory effects of PD-L1 on tumor cells and MDSCs are alleviated, removing key barriers hindering T-cell-mediated anti-tumor responses [189].

Nanomedicine refers to the utilization of nano-preparation technology to either downsize bulk drug substances into nanoscale particles or amalgamate suitable carrier materials with these substances, resulting in the formation of nanoscale particles and final drug preparations. This field has garnered considerable interest due to its potential to markedly enhance efficacy while minimizing non-targeted side effects [190]. miR-122, a liver-specific miRNA, plays a crucial role in preserving liver homeostasis. Past research has identified a correlation between reduced miR-122 expression and the progression of HCC. Moreover, miR-122 exhibits the ability to impede various liver metastases by inhibiting angiogenesis [191–193]. Building upon this foundation, Sendi et al. conducted research by developing a galactose-targeted lipid calcium phosphate (Gal-LCP) nanoformulation of miR-122, enabling the targeted delivery of miR-122 to hepatocytes. The results showed that in various CRCLM models, Gal-LCP miR-122 effectively reduces CRCLM and prolongs survival time. The mechanism behind this effect may be associated with the downregulation of key genes in inflammatory pathways. Additionally, Gal-LCP miR-122 indirectly leads to an increase in the ratio of CD8+/CD4+T cells and a reduction in the infiltration of immunosuppressive cells such as Tregs and MDSCs [194]. Ding et al. devised a nanomedicine encapsulating a cyclin-dependent kinase (CDK) inhibitor and a PD-L1 antibody. This innovative formulation not only triggered the differentiation of dendritic cells (DCs) but also bolstered the infiltration of CD8+T cells. Impressively, this targeted therapeutic strategy demonstrated efficacy in combating LM, highlighting its potential in LM-targeted therapy [195].

Through our analysis, combination therapy has emerged as a notably superior approach compared to single therapy. To refine future treatment programs and guide research efforts, we must focus on comprehensive clinical trials, biomarker discovery, mechanistic elucidation, precision medicine integration, therapeutic innovation, and data-driven optimization. These strategies will facilitate the development of safer and more effective treatments for liver metastases.

## Conclusion

The process of LM involves multiple steps and is influenced by numerous factors both within and outside the liver. While significant progress has been made in the past decade in understanding LM, effective prevention and treatment methods remain limited. Further elucidating the mechanisms by which T cells contribute to the process of LM could undoubtedly provide new insights for research and offer more powerful tools for clinical treatment.
